# Genetic deletion of IL-25 (IL-17E) confers resistance to dextran sulfate sodium-induced colitis in mice

**DOI:** 10.1186/2045-3701-4-72

**Published:** 2014-11-26

**Authors:** An-Jiang Wang, Allen Smith, Yanfei Li, Joseph F Urban, Thirumalai R Ramalingam, Thomas A Wynn, Nonghua Lu, Terez Shea-Donohue, Zhonghan Yang, Aiping Zhao

**Affiliations:** Departments of Radiation Oncology and Medicine, University of Maryland School of Medicine, 10 S. Pine Street, MSTF, Room 7-00D, Baltimore, MD 21201 USA; Department of Gastroenterology and Hepatology, The First Affiliated Hospital of Nanchang University, Nanchang, 330006 China; U.S. Department of Agriculture, Beltsville Human Nutrition Research Center, Agricultural Research Service, Diet, Genomics, and Immunology Laboratory, Beltsville, MD 20705 USA; Immunopathogenesis Section, Laboratory of Parasitic Diseases, National Institute of Allergy and Infectious Diseases, National Institutes of Health, Bethesda, MD 20892 USA; Department of Biochemistry, Zhongshan Medical School, Sun Yat-sen University, 74 Zhongshan 2nd Road, Guangzhou, 510080 China

**Keywords:** IL-25, Colitis, DSS, Mice, IL-13, IL-33

## Abstract

**Background:**

IL-25 is emerging as a key regulator of inflammation in the intestinal mucosa because of its ability to promote type 2 while suppressing Th1 and Th17 responses. Several previous studies reported inconsistent results on the role of exogenous IL-25 in development of colonic inflammation and none were performed in animals with a genetic deletion of IL-25. We investigated the contribution of endogenous IL-25 to DSS-induced colitis using mice deficient in IL-25.

**Results:**

Mice were exposed to DSS in drinking water ad libitum either for seven days (acute) or for three cycles of seven days with DSS followed by 14 days without DSS (chronic) to induce colitis, respectively. The loss of body weight, appearance of diarrhea and bloody stools, and shortening of colon length were significantly less pronounced in IL-25^−/−^ mice compared to WT mice after exposure to acute DSS. Histological examination showed that DSS-treated IL-25^−/−^ mice had only mild inflammation in the colon, while severe inflammation developed in DSS-treated WT mice. A significant up-regulation of IL-33 was observed in acute DSS-treated WT but not in the IL-25^−/−^ mice. There was significantly lower expression of pro-inflammatory cytokines in the colon of acute DSS-treated IL-25^−/−^ compared to WT mice. IL-25^−/−^ mice were also partially protected from chronic DSS challenge especially during the first 2 cycles of DSS exposure. In contrast to IL-25^−/−^ mice, IL-13^−/−^ mice were more susceptible to DSS-induced colitis. Finally, stimulation of T84 colonic epithelial cells with IL-25 up-regulated the expression of IL-33 and several pro-inflammatory cytokines.

**Conclusions:**

These data indicate that endogenous IL-25 acts as a pro-inflammatory factor in DSS-induced colitis, which is unlikely to be mediated by IL-13 but possibly the induction of IL-33 and other pro-inflammatory mediators from colonic epithelial cells. The present study suggests that IL-25 may contribute to the pathogenesis of inflammatory bowel disease in at least a subgroup of patients.

Inflammatory bowel disease (IBD), a term that includes both ulcerative colitis (UC) and Crohn’s disease (CD), is characterized by chronic relapsing inflammation of the intestine leading to diarrhea and abdominal pain. It is estimated that some 1.4 million Americans suffer from IBD, with approximately 30,000 new cases diagnosed each year [1]. There is no cure for IBD and the relapsing course of the disease requires extended patient care with progressive medical or surgical interventions to induce remission [2]. Patients with long-standing colitis also have an increased risk of developing colorectal cancer. Although it is generally believed that IBD results from the combination of alterations in the intestinal microbiome, a genetic predisposition, and a dysregulated immune response, the exact etiological factors and underlying molecular mechanisms for the pathogenesis of IBD remain to be fully elucidated [3–5]. Excessive cytokine responsiveness is a well recognized pathogenic factor important for IBD. For example, patients with CD are often associated with an exaggerated production of IFNγ, TNFα, and other Th1-derived cytokines. Ulcerative colitis, on the other hand, is considered to be an atypical Th2-mediated pathology associated with increased production of IL-13 [6, 7]. Nevertheless, increased expression of Th1 and Th17 cytokines are reported in both CD and UC patients [8–12] and anti-TNFα is therapeutically effective for both types of IBD patients.

IL-25, also called IL-17E, is a member of IL-17 cytokine family that includes IL-17A-F. While other members of the IL-17 family have biological activities similar to Th1 inflammatory cytokines, IL-25 is involved in the promotion of type 2 immunity including allergy, asthma, and host immunity against parasitic nematode infection. In addition, IL-25 is capable of inhibiting pro-inflammatory Th1 and Th17 cytokine responses that are implicated in various types of autoimmune diseases [13]. Mice with IL-25 deficiency (IL-25^−/−^) develop severe intestinal inflammation during nematode infection implicating a pivotal role of IL-25 in gut mucosal homeostasis [14, 15]. In fact, both up-regulation and down-regulation of the IL-25 expression in the colonic mucosa were reported in patients with IBD or experimental murine models of colitis [16, 17]. Several studies also showed that exogenous IL-25 either ameliorates or aggravates colitis in mice depending on the disease model of colitis employed ([6, 18]. Notably, none of those studies were performed in mice with a genetic deletion of IL-25, and therefore, a role for endogenous IL-25 in the development of colonic inflammation remains to be elusive.

Given that IL-25 has potent immune modulating activities especially in innate immunity and that dysregulated innate immunity plays a dominant role in pathogenesis of IBD, the current study investigated whether endogenous IL-25 contributes to the development of colonic inflammation using a model of dextran sulfate sodium (DSS)-induced colitis in mice. Results from this study showed reduced colitis in mice genetically lacking IL-25 after exposure to DSS, thus providing evidence that endogenous IL-25 may have a pro-inflammatory role in the development of colonic inflammation.

## Results

### Mice deficient in IL-25 are partially protected from dextran sulfate sodium-induced acute and chronic colitis

**Figure 1:**
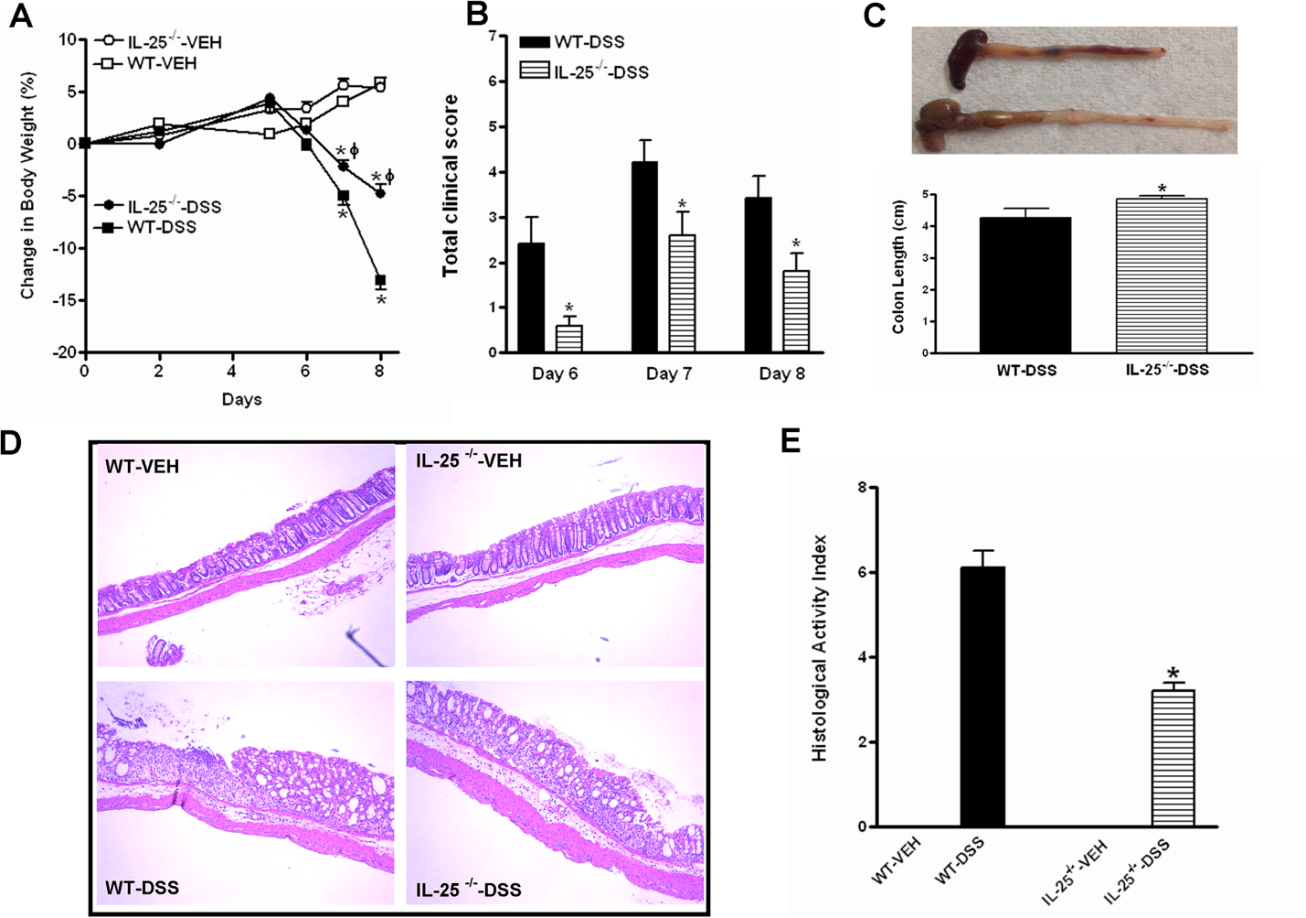
**Mice genetically deficient in IL-25 are protected from acute dextran sulfate sodium (DSS)-induced colitis.** Mice were given 3.2% DSS in drinking water for 7 days and euthanized at day 8. **(A)** Changes in body weight during the course of experiment, expressed as the percentage of initial body weight at day 0; **(B)** Clinical disease activity based on stool consistency (0–3), presence of blood in stool (0–2), and general appearance (0–2); **(C)** Colon length at euthanasia; **(D)** Representative H&E-stained colon sections (100X); **(E)** Histological activity index from a total score of epithelial damage (0–4) and inflammatory cell infiltration (0–4). Data are mean ± SEM and are representatives of three independent experiments with five mice per group. *P < 0.05 versus respective WT-DSS.

To examine the role of endogenous IL-25 in the development of DSS-induced acute colitis, WT and IL-25^−/−^ mice received DSS in the drinking water ad libitum for seven days and were euthanized on day eight. When compared to WT mice, IL-25^−/−^ mice lost significantly less body weight (Figure 1A) and had less severe disease activity based on combined clinical scores of stool consistency, blood in the stool, and mouse appearance during the course of DSS exposure (Figure 1B). Both strains of mice on DSS had shortened colon lengths as compared to the vehicle-treated controls, however, the shortening in IL-25^−/−^ mice was significantly less than that of DSS-treated WT mice (Figure 1C). Histological evaluation of H&E-stained tissue sections of colon showed that IL-25^−/−^ mice treated with DSS had lower HAI with only mild inflammation, while extensive inflammation developed in DSS-treated WT mice based on the extent of focal crypt lesions, loss of goblet cells, and infiltration of inflammatory cells (Figure 1D & E). These results suggest that endogenous IL-25 plays a pro-inflammatory role in DSS-induced acute colitis, rather than an anti-inflammatory role proposed by a previous study using exogenous IL-25 [19, 20].

**Figure 2:**
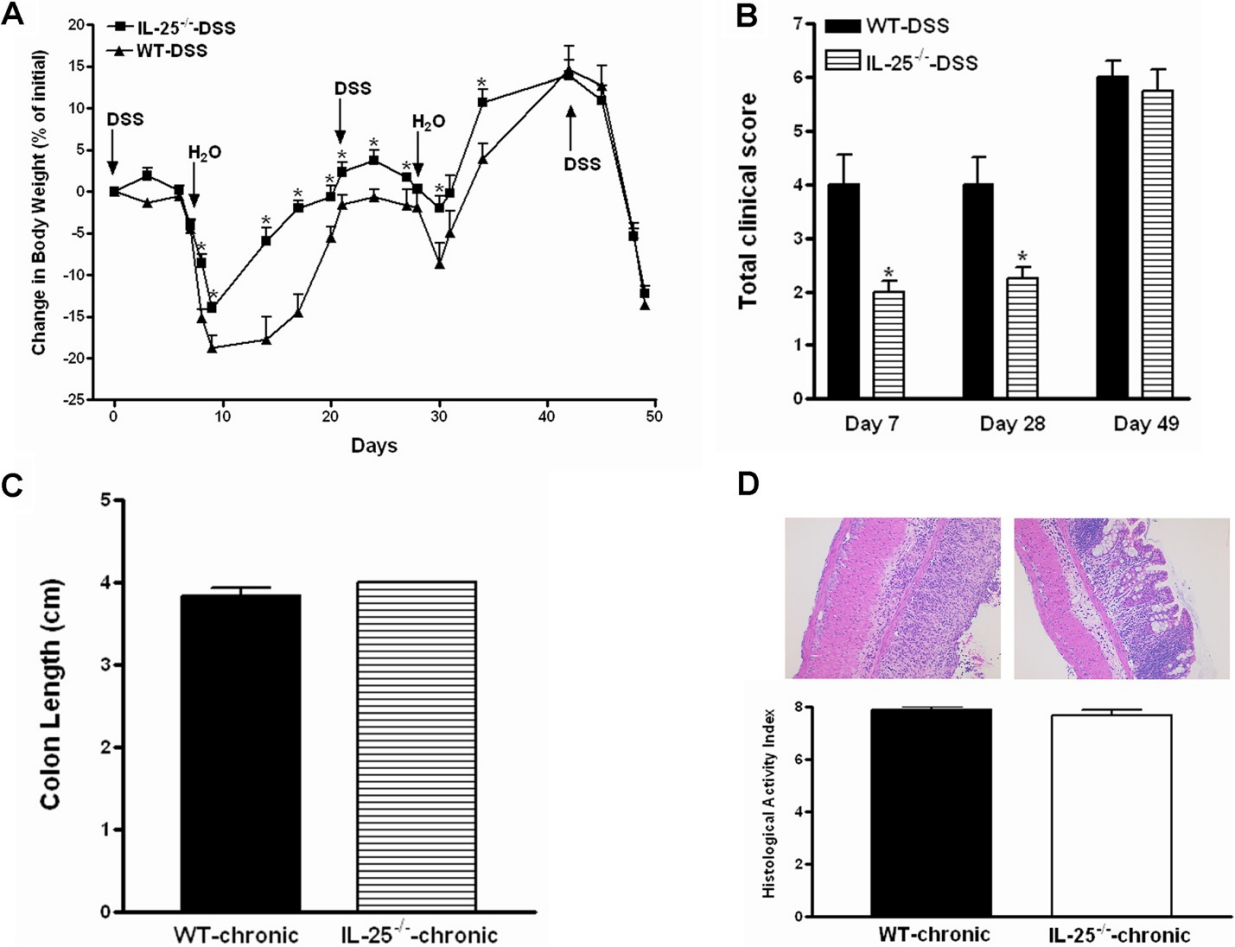
**Mice genetically deficient in IL-25 are partially protected from chronic dextran sulfate sodium (DSS)-induced colitis.** Mice were exposed to three cycles of DSS treatment. Each cycle of DSS involved seven days of DSS followed by 14 days on regular water. Mice were euthanized at the eighth day of the third DSS cycle. **(A)** Body weight change expressed as the percentage of initial body weight at day 0; **(B)** Clinical disease activity based on stool consistency (0–3), presence of blood in stool (0–2), and general appearance (0–2); **(C)** Colon length at euthanasia; **(D)** Representative H&E-stained colon sections (100X) and the histological activity index from a total score of epithelial damage (0–4) and inflammatory cell infiltration (0–4). Data are mean ± SEM and are representatives of two independent experiments with five or six mice per group. *P < 0.05 versus the respective WT.

We next utilized a model of chronic DSS-induced colitis to further evaluate the role of IL-25. As expected, both WT and IL-25^−/−^ mice lost body weight compared to vehicle-treated controls, but gained body weight when DSS treatment was interrupted. Notably, IL-25^−/−^ mice lost significantly less body weight during the first two cycles of DSS exposure and recovered faster after the switch back to water compared to DSS-treated WT mice (Figure 2A). In addition, lower disease activity scores were recorded in IL-25^−/−^ mice than in WT mice when examined at the end of first (day 7) and second (day 28) cycle of DSS exposure (Figure 2B). However, as the third cycle of DSS began, both IL-25^−/−^ mice and WT mice lost body weight in a similar more rapid rate than observed in the first two cycles of DSS exposure. No differences in weight loss between the DSS-treated IL-25^−/−^ and WT mice were detected during the third cycle of DSS exposure, and the total clinical scores were similar at day 49 (Figure 2B). At euthanasia, both IL-25^−/−^ and WT mice treated with DSS had a similar shortening of colon length (Figure 2C), and microscopic examination showed complete loss of crypts as well as extensive inflammatory cell infiltration in the muscularis mucosa, submucosa, and muscularis externa (Figure 2D). Dramatic and similar smooth muscle hyperplasia/hypertrophy was also evident in both IL-25^−/−^ and WT mice. These results are consistent with the notion that mice genetically deficient in IL-25 are only partially protected from DSS-induced chronic colitis.

### Associated changes in cytokine profile of the colon

**Figure 3:**
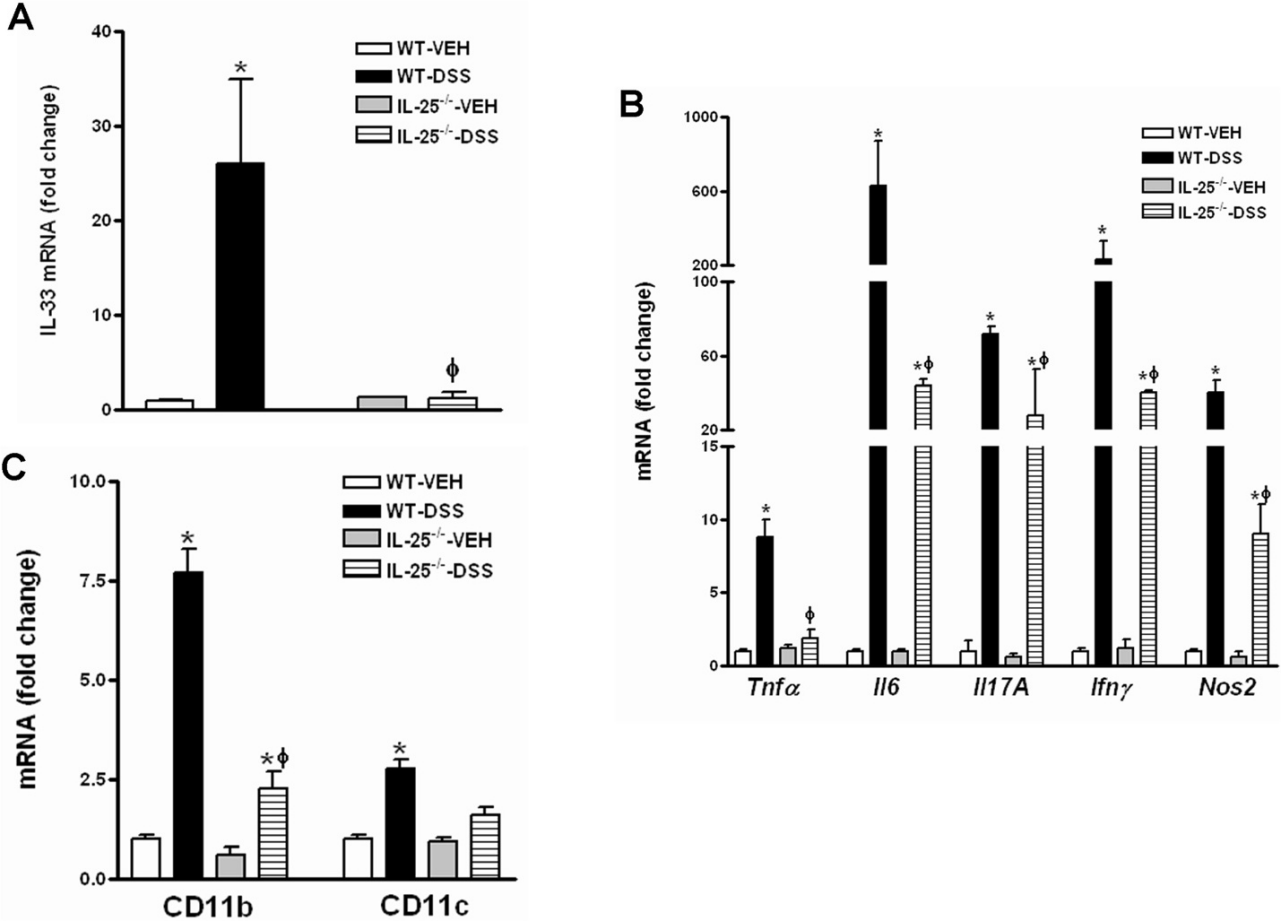
**Colonic expression of cytokines, mediators, and inflammatory cell markers.** Mice genetically deficient in IL-25 and WT mice were given 3.2% DSS in drinking water for 7 days and euthanized at day 8. qPCR was performed to examine mRNA expression of IL-33 **(A)**, pro-inflammatory cytokines/mediators **(B)**, and inflammatory cell markers **(C)** in the colon. The fold changes are relative to the individual WT-VEH groups after normalization to 18 s rRNA. Data are mean ± SEM and are representatives of two independent experiments with five mice per group. *P < 0.05 versus the respective VEH; ϕ P < 0.05 versus respective WT-DSS.

To explore the potential mechanisms underlying the relative protection of IL-25^−/−^ mice against DSS-induced colitis, we evaluated the immune response in colonic tissues collected from mice exposed to acute or chronic DSS treatment. qPCR analysis showed a significant up-regulation of IL-33 in the colon of acute DSS-treated WT mice (Figure 3A), a type 2-associated cytokine that reportedly contributes to intestinal inflammation [21, 22]. However, the up-regulation of IL-33 was virtually absent in acute DSS-treated IL-25^−/−^ mice (Figure 3A). Significantly increased levels of pro-inflammatory cytokines/mediators TNFα, IL-6, IL-17A, IFNγ, and NOS2 were also observed in the colons of both WT and IL-25^−/−^ mice post acute DSS treatment (Figure 3B). However, the fold change in expression of these cytokines/mediators was significantly lower in IL-25^−/−^ mice when compared to WT mice (Figure 3B). The acute DSS-induced up-regulation of expression for CD11b and CD11c, markers of inflammatory cell infiltrates, was also less pronounced in the colons of IL-25^−/−^ than WT mice (Figure 3C). However, we were unable to detect significant differences in the colonic expression of pro-inflammatory cytokine/mediators between WT and IL-25^−/−^ mice when the samples were collected at the end of three cycles of chronic DSS treatment (data not shown). This is consistent with the observation that protection against DSS colitis in IL-25^−/−^ mice was lost by the third cycle of DSS treatment.

### Genetic deletion of IL-13 in mice increases susceptibility to dextran sulfate sodium-induced colitis

**Figure 4:**
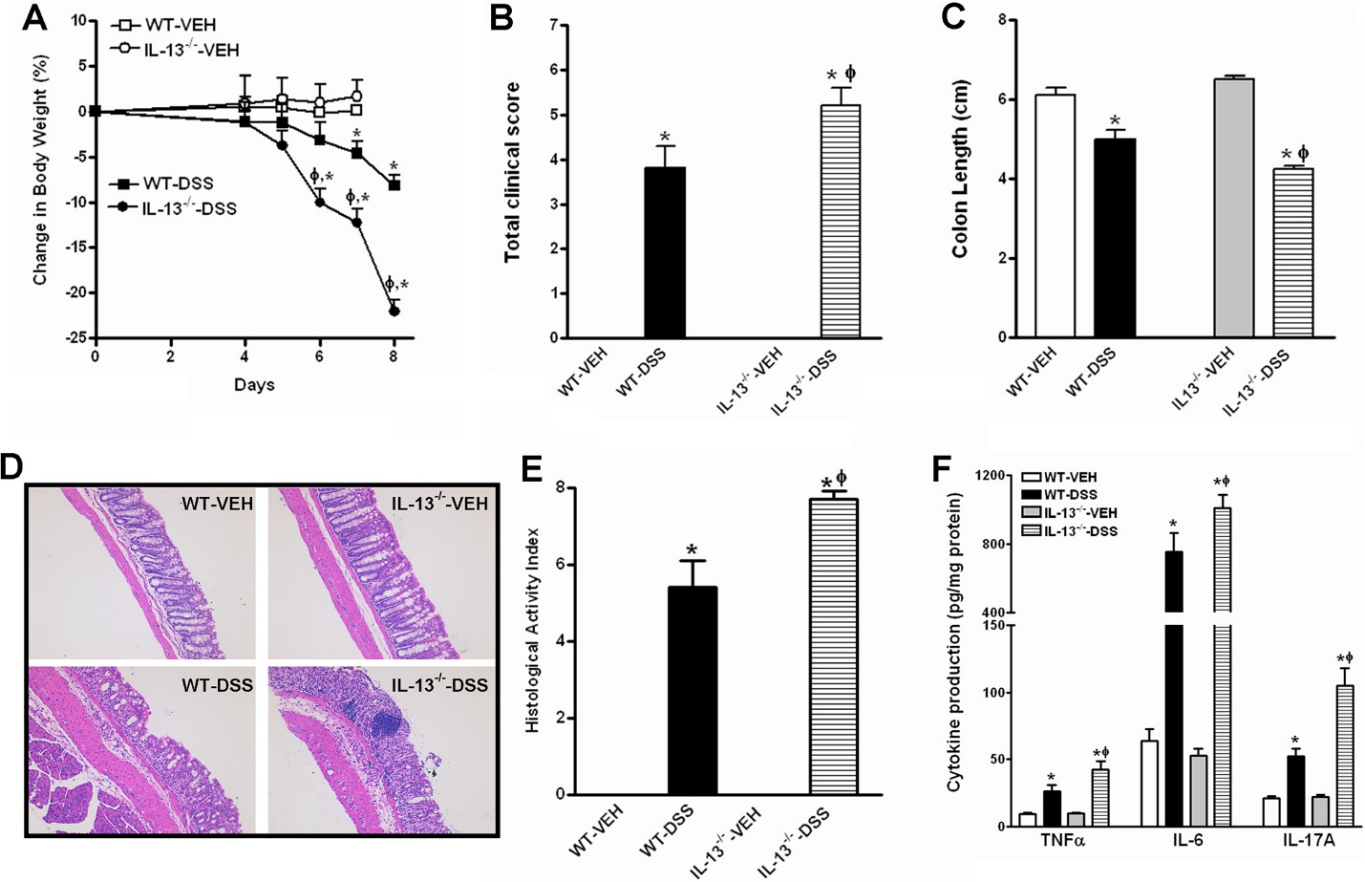
**Mice genetically deficient in IL-13 are more susceptible to acute dextran sulfate sodium (DSS)-induced colitis.** Mice were given 3.0% DSS in drinking water for 7 days and euthanized at day 8. **(A)** Body weight change expressed as the percentage of initial body weight at day 0; **(B)** Clinical disease activity based on stool consistency (0–3), presence of blood in stool (0–2), and general appearance (0–2); **(C)** Colon length at euthanasia; **(D)** Representative H&E-stained colon sections (100X); **(E)** Histological activity index from a total score of epithelial damage (0–4) and immune cell infiltration (0–4); **(F)** In situ cytokine production in the colon by ELISA. Data are mean ± SEM and are representatives of two independent experiments with five mice per group. *P < 0.05 versus the respective VEH; ϕ P < 0.05 versus respective WT.

IL-13 is one of the major downstream effector molecules that mediates the biological activities of IL-25 [23]. To address whether resistance to DSS-induced colitis conferred by IL-25 deficiency was attributable to a defect in IL-13 expression, we exposed IL-13^−/−^ mice to DSS for seven days. When compared with the age- and sex-matched WT mice, IL-13^−/−^ mice lost significantly more body weight (Figure 4A) and developed exacerbated disease activity (Figure 4B) during the course of acute DSS exposure, contrary to that observed in IL-25^−/−^ mice. At euthanasia, the extent of colon shortening was worse in DSS-treated IL-13^−/−^ mice than in WT mice (Figure 4C). In addition, the colons from DSS-treated IL-13^−/−^ mice had significantly higher HAI than that of DSS-treated WT mice, evidenced by the presence of severe denudation of the surface epithelium, extensive loss of crypts and separation of the crypt base from the muscularis mucosa, as well as enhanced inflammatory cell infiltration in the submucosa (Figure 4D & E). ELISA analysis of in situ cytokine production revealed that the colons from DSS-treated IL-13^−/−^ mice produced significantly more IL-6, TNFα, and IL-17A than that of DSS-treated WT mice (Figure 4F). Overall, these results suggest that mice deficient in IL-13 have increased susceptibility to acute DSS-induced colitis, ruling out the possibility that the decreased susceptibility to DSS-induced colitis in IL-25^−/−^ mice is due to a lack of IL-13.

### Induction of pro-inflammatory cytokines/chemokines in colonic epithelial cells by IL-25

**Figure 5:**
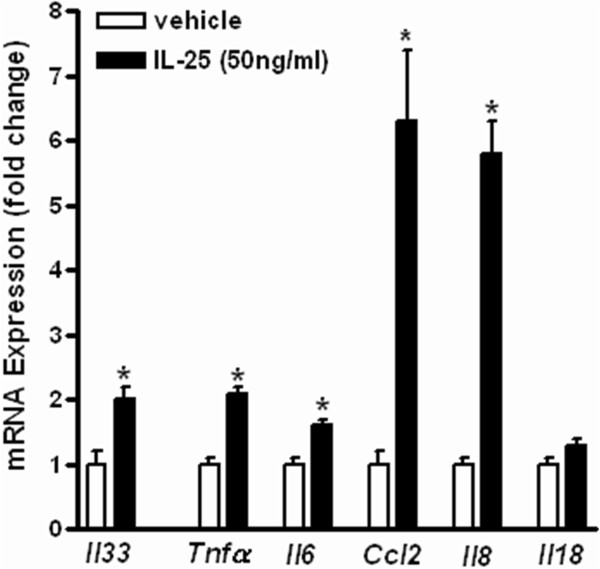
**IL-25 induces up-regulation of cytokines in T84 colonic epithelial cells.** T84 cells were cultured in a mixture of Ham’s F12 and Dulbecco’s modified Eagle’s medium containing 5% FBS. One million cells per well were seeded on a 6-well plate and incubated overnight, and then treated with IL-25 (50 ng/ml) for 24 hours in triplicates. Cells were collected for mRNA expression of cytokines by qPCR. Data are mean ± SEM and are representatives of two independent experiments. *P < 0.05 versus the respective vehicle.

Dysregulated function and immune response of the intestinal epithelium are important contributors to intestinal inflammation. A previous study showed that epithelial cells produce and respond to IL-25 [24]. We cultured T84 cells, a human colonic epithelial cell line, to determine whether IL-25 directly stimulates epithelial cells to produce pro-inflammatory cytokines that may contribute to colitis. After stimulation with IL-25 for 24 hours, T-84 cells expressed significantly higher levels of IL-33 (Figure 5). The results are consistent with in vivo results showing that mice deficient in IL-25 produced less IL-33 in the colon (Figure 3A). In addition, IL-25 induced up-regulation of pro-inflammatory cytokines TNFα and IL-6 as well as chemokines CCL2 and IL-8, but did not affect the expression of IL-18 in T84 cells (Figure 5).

## Discussion

IL-25 is emerging as a key regulator of inflammation in the intestinal mucosa because of its ability to promote type 2 while suppressing Th1 and Th17 cytokine responses. The role of IL-25 in the development of colonic inflammation, however, remained to be fully elucidated. As a primarily epithelial-derived cytokine in the gastroenterological (GI) tract, IL-25 is an important player in control of intestinal immunity and function. Exogenous IL-25 induces strong type 2 immunity in the GI tract characterized by increased expression of type 2-related cytokines such as IL-5 and IL-13 [25, 26], whereas mice deficient in IL-25 have impaired ability to expel parasitic worms and develop severe intestinal inflammation in response to enteric nematode infection [15, 26]. IL-25 is also involved in the regulation of intestinal function including smooth muscle contraction, epithelial cell secretion, as well as epithelial barrier function [26]. Recent studies further identified type 2 innate lymphoid cells as the key IL-25-responsive cells that produce IL-5 and IL-13, major downstream effector molecules of IL-25 in promotion of type 2 immunity [27]. There are, however, inconsistent reports regarding the role of IL-25 in the development of colonic inflammation. Caruso et al. reported that patients with either CD or UC had significantly less IL-25 produced in the colon, implying an anti-inflammatory role of IL-25 [16]. Of note, patients with IBD are often accompanied by severe loss of intestinal epithelium, which may account for the decrease in IL-25 levels since it is expressed exclusively by epithelial cells in the GI tract [16]. The same report also showed that exogenous IL-25 ameliorated mouse TNBS colitis that features up-regulation of pro-inflammatory Th1 cytokines as seen in CD, or oxazolone colitis that has histological characteristics and elevated production of type 2 cytokines resembling UC patients [16]. On the other hand, Camelo et al. showed that IL-25 mRNA was modestly up-regulated in the colonic epithelial cells of the mice during oxazolone colitis, and blocking IL-25 signaling via neutralizing IL-25 or IL-17RB considerably improved the clinical aspects of the colitis [17]. Our current study demonstrated that mice genetically deficient in IL-25 were partially protected from DSS-induced colitis, a model with a Th1/Th17 dominant response at the early acute stage that shifts to a type 2 response in the chronic stage [28]. This protection was observed in both acute and chronic disease models, albeit with a loss in protection over time after a third round of DSS treatment. The relative resistance to colitis resulted from IL-25 deficiency was evidenced by significantly decreased disease activity as well as histological activity index, and was associated with reduction of various pro-inflammatory cytokines/mediators in the colon. Our results are contrary to a previous study showing that exogenous IL-25 had an inhibitory effect on the development of DSS colitis [19]. The reason for the inconsistency is not known, but a follow-up study from the same group indicated that only high doses (0.8 μg daily) of exogenous IL-25 protected, while low doses (0.2 μg daily) of IL-25 aggravated the DSS-induced colitis [20]. We made attempts to replicate those studies with exogenous IL-25 [19, 20], but the results were inconclusive since daily IL-25 administration significantly reduced water/DSS consumption of the mice, which altered the outcome (Zhao A, et al. unpublished results). It is possible that the decrease in DSS consumption actually accounted for the protective effects of exogenous IL-25 observed in the previous studies [19, 20]. While none of the previous studies were performed to understand the role of endogenous IL-25 in mouse models of colitis, our results demonstrate that constitutive IL-25 is a pro-inflammatory factor for the development of DSS colitis in mice and, therefore, may contribute to colonic inflammation in at least a subgroup of patients with IBD.

IL-33 is a recently identified member of the IL-1 cytokine family and is expressed by many types of cell ranging from major innate immune cells to non-hematopoietic structural cells throughout the GI tract [29, 30]. Similarly to IL-25, epithelial-derived IL-33 is thought to be important in initiating type 2 immunity especially in the intestine and lung [31]. A role of IL-33 for colonic inflammation has been suggested based on several recent studies [32]. Expression of IL-33 and its ST2 receptor was consistently up-regulated in the intestinal mucosa of patients with UC and to a lesser extent in CD patients, as well as animals with colitis [22, 33]. In addition, ST2 deficiency led to decreased disease severity in TNBS- and DSS-induced mouse models of colitis whereas exogenous IL-33 exacerbated DSS-colitis [22]. We and others showed that both IL-25 and IL-33 can impair intestinal epithelial barrier function that may potentiate inflammation [22, 26, 34]. In the current study, we replicated previous results [21, 22] that showed up-regulation of IL-33 in DSS-induced colitis and, more importantly, demonstrated that resistance of IL-25^−/−^ mice to DSS-induced colitis was associated with diminished IL-33 expression in the colon. These results suggest that IL-25 may activate IL-33 signaling leading to heightened inflammation. Indeed, both epithelial cells and lamina propria immune cells in the GI tract express the IL-17RB and IL-17RA receptor subunits for IL-25 that are regulated by immune mediators including IL-25 [26], and exogenous administration of IL-25 to mice increases the expression of IL-33 in the intestine [24]. We showed previously that exogenous IL-33 also up-regulated the expression of IL-25 [34]. These results suggest that there is a positive feedback loop between IL-25 and IL-33 that potentiates the biological activities. Our current results extend this observation by showing that IL-25 can directly act on a cell line of colonic epithelial cells to induce IL-33 expression.

Among the identified downstream effector molecules of IL-25, IL-13 is critical for the promotion of type 2 immunity important for clearance of parasitic nematode infections and for the characteristic changes in gut function after nematode infection (35). Indeed, exogenous IL-25 could not restore the host protective immunity or hyper-contractility in IL-13^−/−^ mice after Nippostrongylus brasiliensis infection [35]. IL-13 appears to also play a role in the pathogenesis of IBD especially UC, since levels of IL-13 were increased in patients with UC [36] and blockage of IL-13 was effective in treatment of murine colitis induced by oxazolone [37]. The observed resistance to DSS-induced colitis in IL-25^−/−^ mice led us to investigate whether this was related to defective IL-13 expression. In the present study, however, mice with a genetic IL-13 deficiency had significantly more severe colitis after exposure to DSS contrary to that observed in IL-25^−/−^ mice, indicating an anti-inflammatory effect of IL-13. Since IL-13 acts almost exclusively through intracellular STAT6 signaling pathway for its biological activities [38], our results are consistent with a previous study showing that DSS-induced colitis was exacerbated in mice deficient in STAT6 because the defective IL-13 signaling led to a higher level of IFNγ [39]. On the other hand, our results from IL-13^−/−^ mice on a C57BL/6 background are contrary to a recent study using IL-13^−/−^ mice on a BALB/c background that showed reduced severity of DSS-induced colitis accompanied by decreased number of enterochromaffin cell and colonic serotonin content [40]. It is known that mice on a C57BL/6 background are prone to Th1 immunity, whereas mice on BALB/c background are biased toward type 2 immunity. Whether the differences in genetic background contribute to the disparate results remains to be determined.

Recent studies highlight the important role of defective epithelial barrier function and mucosal immunity in the development of IBD [41]. Intestinal epithelial cells that line the surface of the gut serve as the initial barrier to prevent antigens from penetrating the intestinal mucosa. Defects in the intestinal epithelial barrier allow the direct exposure of mucosa immune cells to harmful luminal contents, resulting in the production of pro-inflammatory cytokines that are implicated in the pathogenesis of IBD. In addition, intestinal epithelial cells produce cytokines and chemokines that regulate the recruitment of inflammatory cells. Therefore, intestinal epithelial cells orchestrate the mucosal immune homeostasis and are one of the major players in the development of intestinal inflammation [41]. Among the cytokines/chemokines produced by epithelial cell, TNFα and IL-6 are considered to be central to the pathogenesis of IBD while CCL2 and IL-8 are the major players for the recruitment of inflammatory cells. Our current study showed that direct stimulation of a cell line of colonic epithelial cells with IL-25 increased expression of IL-6, TNFα, as well as IL-8 and CCL2, providing a potential mechanism by which IL-25 contributes to DSS-induced colitis. Together with their ability to produce IL-33 in response to IL-25, intestinal epithelial cells are likely an important player mediating the pro-inflammatory role of IL-25 in DSS-induced colitis.

## Conclusions

Type 2 immunity is essential for host protection against parasitic nematode infection while is detrimental in certain type 2-mediated inflammatory pathologies. Our present study shows that mice deficient in IL-25 are partially protected from DSS-induced colitis in mice associated with lower expression of IL-33 and other pro-inflammatory cytokines/mediators in the colon. In contrast, mice deficient in IL-13 are more susceptible to the colitis indicating the protection conferred by IL-25 deficiency is unlikely due to a lack of IL-13. Since IBD is a heterogeneous group of chronic inflammatory disorders of the GI tract in that various immune factors are implicated, the current study provides genetic evidence for the first time that constitutively expressed IL-25 may contribute to the pathogenesis of IBD in at least a subgroup of patients.

## Methods

### Mice

C57BL/6 wild type (WT) mice were purchased from National Cancer Institute Mouse Repository (Frederick, MD 21702). Mice deficient in IL-25 (IL-25^−/−^) were generated in a mixed background by Regeneron Pharmaceuticals (Tarrytown, NY) [26]. Mice deficient in IL-13 (IL-13^−/−^) were generated in a mixed background [42] and managed by Taconic at the National Institutes of Allergy and Infectious Diseases. These mice were backcrossed to C57BL/6 mice for at least 10 generations after being received, which is considered fully congenic and therefore C57BL/6 WT mice were used as the controls. To avoid the potential confounding effects from differences in the colonic microbiome, all mice with expanded numbers were bred in the USDA/Beltsville animal facility so that they were expected to have the same level of environmental exposure to microbes. All mice were used at the age of 10 to 14 week old and all studies were conducted with institutional approval from both the University of Maryland, Baltimore and the USDA Beltsville Area Animal Care and Use Committees (protocol #13-003) in accordance with principles set forth in the Guide for Care and Use of Laboratory Animals, Institute of Laboratory Animal Resources, National Research Council, Health and Human Services Publication (National Institutes of Health 85–23, revised 1996).

### Induction of DSS colitis in mice

To induce acute colitis, mice (n = 5/group) were administrated 3.0 or 3.2% DSS (mol. wt. 35,000 to 50,000 Da; MP Biochemicals, Santa Ana, CA) in their drinking water ad libitum for seven days and euthanized on day eight. For induction of chronic colitis, mice were exposed to three cycles of 3.2% DSS treatment for seven days each followed by a 14 day interval on regular water [43]. Mice were euthanized one day after the last DSS treatment. Mice on regular drinking water were used as vehicle controls. Age- and sex-matched WT and genetically deficient mice were used throughout the studies. The baseline body weight was not significantly different among the groups of WT, IL-25^−/−^, and IL-13^−/−^ mice when the treatments started.

### Clinical scoring and histological assessment of colitis

During the course of experiments, mice were scored for stool consistency (0–3), presence of blood in stool (0–2), and general appearance (0–2) according to the published “Clinical Scoring Criteria” [43]. These scores were added to generate a total clinical score ranging from 0 to 7. When mice were euthanized, the entire colon was quickly removed and the length of the colon was measured. Segments of distal colon were fixed in 4% paraformaldehyde for 4 hours. Tissue sections (4 μm) of distal colon were cut from paraffin-embedded blocks and stained with hematoxylin and eosin (H&E). Histological assessment of colitis was performed by an investigator who was unaware of the mouse genotype and treatment using established criteria with slight modification [28, 44]. Histological activity index (HAI) was a total score of epithelial damage (0–4) and inflammatory cell infiltration (0–4) ranging from 0 to 8 [28, 44].

### T84 cell culture

T84 cells (CCL-248, ATCC), a human colonic epithelial cell line, were cultured in a mixture of Ham’s F12 and Dulbecco’s modified Eagle’s medium containing 5% FBS. 1 × 10^6^ cells per well were seeded in a 6-well plate and incubated at 37°C with 5% CO_2_ overnight. The cell monolayer was then treated with recombinant IL-25 (50 ng/ml) from Biolegend (San Diego, CA 92121) for 24 hours. After treatment, cell monolayers were collected with Trizol for subsequent RNA isolation and cytokine expression analysis by qPCR.

### RNA extraction, cDNA synthesis, real-time quantitative PCR (qPCR), and ELISA

Total RNA was extracted from cells and tissues with TRIzol reagent (Invitrogen, Grand Island, NY) as per the manufacturer’s instructions. One-centimeter of distal colon was collected and the whole tissue was processed for RNA isolation. RNA samples (2 μg) were reverse-transcribed to cDNA using the First Strand cDNA Synthase Kit (MBI Fermentas, Hanover, MD) with random hexamer primer. qPCR was performed on a CFX96 Touch Real-Time Detection System (Bio-Rad, CA) in a 25 μl volume using SYBR green Supermix (Bio-Rad, Hercules, CA). Amplification conditions were: 95°C for 3 min, 50 cycles of 95°C for 15 s, 60°C for 15 s, and 72°C for 20s. The fold-changes in mRNA expression for targeted genes were relative to the respective groups of vehicle-treated mice after normalization to 18 s rRNA. Tissue homogenates of colonic whole tissues were prepared with RIPA buffer (Cell signaling technology, Beverly, MA) and in situ cytokine production of TNFα, IL-6, and IL-17A in the homogenates was measured using LEGEND MAX™ Mouse ELISA Kit (BioLegend, San Diego, CA) following manufacturer's instructions.

### Data analysis

Statistical analysis was performed using one-way ANOVA followed by Neuman-Keuls test to compare the difference among three or more treatment groups or the Student t test to compare the difference between two groups. P values of <0.05 were considered significant.

## References

[1] Hanauer SB (2006). Inflammatory bowel disease: epidemiology, pathogenesis, and therapeutic opportunities. Inflamm Bowel Dis.

[2] Targan SR (2006). Current limitations of IBD treatment: where do we go from here?. Ann N Y Acad Sci.

[3] Bamias G, Cominelli F (2007). Immunopathogenesis of inflammatory bowel disease: current concepts. Curr Opin Gastroenterol.

[4] Cobrin GM, Abreu MT (2005). Defects in mucosal immunity leading to Crohn's disease. Immunol Rev.

[5] Pizarro TT, Cominelli F (2007). Cytokine therapy for Crohn's disease: advances in translational research. Annu Rev Med.

[6] Fuss IJ, Heller F, Boirivant M, Leon F, Yoshida M, Fichtner-Feigl S, Yang Z, Exley M, Kitani A, Blumberg RS, Mannon P, Strober W (2004). Nonclassical CD1d-restricted NK T cells that produce IL-13 characterize an atypical Th2 response in ulcerative colitis. J Clin Invest.

[7] Heller F, Florian P, Bojarski C, Richter J, Christ M, Hillenbrand B, Mankertz J, Gitter AH, Burgel N, Fromm M, Zeitz M, Fuss I, Strober W, Schulzke JD (2005). Interleukin-13 is the key effector Th2 cytokine in ulcerative colitis that affects epithelial tight junctions, apoptosis, and cell restitution. Gastroenterology.

[8] Fuss IJ, Neurath M, Boirivant M, Klein JS, de la Motte C, Strong SA, Fiocchi C, Strober W (1996). Disparate CD4+ lamina propria (LP) lymphokine secretion profiles in inflammatory bowel disease. Crohn's disease LP cells manifest increased secretion of IFN-gamma, whereas ulcerative colitis LP cells manifest increased secretion of IL-5. J Immunol.

[9] Schmidt C, Giese T, Ludwig B, Mueller-Molaian I, Marth T, Zeuzem S, Meuer SC, Stallmach A (2005). Expression of interleukin-12-related cytokine transcripts in inflammatory bowel disease: elevated interleukin-23p19 and interleukin-27p28 in Crohn's disease but not in ulcerative colitis. Inflamm Bowel Dis.

[10] Fujino S, Andoh A, Bamba S, Ogawa A, Hata K, Araki Y, Bamba T, Fujiyama Y (2003). Increased expression of interleukin 17 in inflammatory bowel disease. Gut.

[11] Nielsen OH, Kirman I, Rudiger N, Hendel J, Vainer B (2003). Upregulation of interleukin-12 and −17 in active inflammatory bowel disease. Scand J Gastroenterol.

[12] Zhang Z, Zheng M, Bindas J, Schwarzenberger P, Kolls JK (2006). Critical role of IL-17 receptor signaling in acute TNBS-induced colitis. Inflamm Bowel Dis.

[13] Owyang AM, Zaph C, Wilson EH, Guild KJ, McClanahan T, Miller HR, Cua DJ, Goldschmidt M, Hunter CA, Kastelein RA, Artis D (2006). Interleukin 25 regulates type 2 cytokine-dependent immunity and limits chronic inflammation in the gastrointestinal tract. J Exp Med.

[14] Kleinschek MA, Owyang AM, Joyce-Shaikh B, Langrish CL, Chen Y, Gorman DM, Blumenschein WM, McClanahan T, Brombacher F, Hurst SD, Kastelein RA, Cua DJ (2007). IL-25 regulates Th17 function in autoimmune inflammation. J Exp Med.

[15] Zaph C, Du Y, Saenz SA, Nair MG, Perrigoue JG, Taylor BC, Troy AE, Kobuley DE, Kastelein RA, Cua DJ, Yu Y, Artis D (2008). Commensal-dependent expression of IL-25 regulates the IL-23-IL-17 axis in the intestine. J Exp Med.

[16] Caruso R, Sarra M, Stolfi C, Rizzo A, Fina D, Fantini MC, Pallone F, MacDonald TT, Monteleone G (2009). Interleukin-25 inhibits interleukin-12 production and Th1 cell-driven inflammation in the Gut. Gastroenterology.

[17] Camelo A, Barlow J, Drynan L, Neill D, Ballantyne S, Wong S, Pannell R, Gao W, Wrigley K, Sprenkle J, McKenzie A (2012). Blocking IL-25 signalling protects against gut inflammation in a type-2 model of colitis by suppressing nuocyte and NKT derived IL-13. J Gastroenterol.

[18] Monteleone G, Pallone F, MacDonald TT (2010). Interleukin-25: a two-edged sword in the control of immune-inflammatory responses. Cytokine Growth Factor Rev.

[19] Mchenga SSS, Wang D, Li C, Shan F, Lu C (2008). Inhibitory effect of recombinant IL-25 on the development of dextran sulfate sodium-induced experimental colitis in mice. Cell Mol Immunol.

[20] Salum Mchenga SS, Wang D, Janneh FM, Feng Y, Zhang P, Li Z, Lu C (2010). Differential dose effects of recombinant IL-25 on the development of dextran sulfate sodium-induced colitis. Inflamm Res.

[21] Pushparaj PN, Li D, Komai-Koma M, Guabiraba R, Alexander J, McSharry C, Xu D (2013). Interleukin-33 exacerbates acute colitis via interleukin-4 in mice. Immunology.

[22] Sedhom MA, Pichery M, Murdoch JR, Foligne B, Ortega N, Normand S, Mertz K, Sanmugalingam D, Brault L, Grandjean T, Lefrancais E, Fallon PG, Quesniaux V, Peyrin-Biroulet L, Cathomas G, Junt T, Chamaillard M, Girard JP, Ryffel B (2013). Neutralisation of the interleukin-33/ST2 pathway ameliorates experimental colitis through enhancement of mucosal healing in mice. Gut.

[23] Fallon PG, Ballantyne SJ, Mangan NE, Barlow JL, Dasvarma A, Hewett DR, McIlgorm A, Jolin HE, McKenzie AN (2006). Identification of an interleukin (IL)-25-dependent cell population that provides IL-4, IL-5, and IL-13 at the onset of helminth expulsion. J Exp Med.

[24] Kang Z, Swaidani S, Yin W, Wang C, Barlow J, Gulen M, Bulek K, Do J, Aronica M, McKenzie A, Min B, Li X (2012). Epithelial cell-specific Act1 adaptor mediates interleukin-25-dependent helminth expulsion through expansion of Lin(−)c-Kit(+) innate cell population. Immunity.

[25] Fort MM, Cheung J, Yen D, Li J, Zurawski SM, Lo S, Menon S, Clifford T, Hunte B, Lesley R, Muchamuel T, Hurst SD, Zurawski G, Leach MW, Gorman DM, Rennick DM (2001). IL-25 induces IL-4, IL-5, and IL-13 and Th2-associated pathologies in vivo. Immunity.

[26] Zhao A, Urban JF, Sun R, Stiltz J, Morimoto M, Notari L, Madden KB, Yang Z, Grinchuk V, Ramalingam TR, Wynn TA, Shea-Donohue T (2010). Critical role of IL-25 in nematode infection-induced alterations in intestinal function. J Immunol.

[27] Licona-Limon P, Kim LK, Palm NW, Flavell RA (2013). TH2, allergy and group 2 innate lymphoid cells. Nat Immunol.

[28] Alex P, Zachos NC, Nguyen T, Gonzales L, Chen TE, Conklin LS, Centola M, Li X (2009). Distinct cytokine patterns identified from multiplex profiles of murine DSS and TNBSΓÇÉInduced colitis. Inflamm Bowel Dis.

[29] Pastorelli L, De Salvo C, Vecchi M, Pizarro TT (2013). The role of IL-33 in gut mucosal inflammation. Mediators Inflamm.

[30] Liew FY, Pitman NI, McInnes IB (2010). Disease-associated functions of IL-33: the new kid in the IL-1 family. Nat Rev Immunol.

[31] Schmitz J, Owyang A, Oldham E, Song Y, Murphy E, McClanahan TK, Zurawski G, Moshrefi M, Qin J, Li X, Gorman DM, Bazan JF, Kastelein RA (2005). IL-33, an interleukin-1-like cytokine that signals via the IL-1 receptor-related protein ST2 and induces T helper type 2-associated cytokines. Immunity.

[32] Salas A (2013). The IL-33/ST2 axis: yet another therapeutic target in inflammatory bowel disease?. Gut.

[33] Beltran CJ, Nunez LE, Diaz-Jimenez D, Farfan N, Candia E, Heine C, Lopez F, Gonzalez MJ, Quera R, Hermoso MA (2010). Characterization of the novel ST2/ILΓÇÉ33 system in patients with inflammatory bowel disease. Inflamm Bowel Dis.

[34] Yang Z, Sun R, Grinchuk V, Blanco JAF, Notari L, Bohl JA, McLean LP, Ramalingam TR, Wynn TA, Urban JF, Vogel SN, Shea-Donohue T, Zhao A (2013). IL-33-induced alterations in murine intestinal function and cytokine responses are MyD88, STAT6, and IL-13 dependent. Am J Physiol Gastrointest Liver Physiol.

[35] Zhao A, McDermott J, Urban JF, Gause W, Madden KB, Yeung KA, Morris SC, Finkelman FD, Shea-Donohue T (2003). Dependence of IL-4, IL-13, and nematode-induced alterations in murine small intestinal smooth muscle contractility on Stat6 and enteric nerves. J Immunol.

[36] Neurath MF (2014). New targets for mucosal healing and therapy in inflammatory bowel diseases. Mucosal Immunol.

[37] Heller F, Fuss IJ, Nieuwenhuis EE, Blumberg RS, Strober W (2002). Oxazolone colitis, a Th2 colitis model resembling ulcerative colitis, is mediated by IL-13-producing NK-T cells. Immunity.

[38] Shea-Donohue T, Fasano A, Smith A, Zhao A (2010). Enteric pathogens and gut function: role of cytokines and STATs. Gut Microbes.

[39] Elrod JW, Laroux SF, Houghton J, Carpenter A, Ando T, Jennings MH, Grisham M, Walker N, Alexander SJ (2005). DSS-induced colitis is exacerbated in STAT-6 knockout mice. Inflamm Bowel Dis.

[40] Shajib M, Wang H, Kim JJ, Sunjic I, Ghia JE, Denou E, Collins M, Denburg JA, Khan WI (2013). Interleukin 13 and serotonin: linking the immune and endocrine systems in murine models of intestinal inflammation. PLoS ONE.

[41] Kaser A, Zeissig S, Blumberg RS (2010). Inflammatory bowel disease. Annu Rev Immunol.

[42] McKenzie GJ, Bancroft A, Grencis RK, McKenzie ANJ (1998). A distinct role for interleukin-13 in Th2-cell-mediated immune responses. Curr Biol.

[43] Maxwell JR, Brown WA, Smith CL, Byrne FR, Viney JL (2009). Methods of inducing inflammatory bowel disease in mice. Curr Protoc Pharmacol.

[44] Obermeief F, Kojouharoff G, Hans W, Scholmerich J, Gross V, Falk W (1999). Interferon-gamma (IFN-gamma)- and tumour necrosis factor (TNF)-induced nitric oxide as toxic effector molecule in chronic dextran sulphate sodium (DSS)-induced colitis in mice. Clin Exp Immunol.

